# Rare But Risky: Clinical Impacts of Pneumothorax After Thyroidectomy and Parathyroidectomy

**DOI:** 10.1002/lary.70646

**Published:** 2026-06-12

**Authors:** Jessan A. Jishu, Alexandra LaForteza, Dara Bruce, Isabel Riccio, Mina Liao, Yaser Y. Bashumeel, Emad Kandil, Mohamed A. Shama

**Affiliations:** ^1^ Tulane University School of Medicine New Orleans Louisiana USA; ^2^ Louisiana State University School of Medicine New Orleans Louisiana USA; ^3^ Department of Surgery Tulane University School of Medicine New Orleans Louisiana USA

**Keywords:** emphysema, endocrine, otolaryngology, pleural injury, retrospective, TriNetX

## Abstract

**Objective:**

Postoperative pneumothorax (PPTX) after thyroidectomy (TD) or parathyroidectomy (PT) is rare and primarily described in case reports with limited data on its prognostic significance in large populations. We aim to assess the incidence of PPTX and its association with clinical outcomes following TD or PT.

**Methods:**

We queried the TriNetX United States Research Network, identifying patients ≥ 18 years of age who underwent open TD or PT between January 2000 and December 2025. Patients were separated by development of PTX within 7 days postoperatively. Outcomes were assessed at 3 and 6 months using odds ratios (ORs) and at 1 year using hazard ratios (HRs), with 95% confidence intervals (CIs). The primary outcome was all‐cause mortality; secondary outcomes included risks for pneumonia, dysphagia, vocal cord paralysis, and need for mechanical ventilation as well as healthcare utilization rates.

**Results:**

Among 228,340 patients undergoing surgery, 237 (0.10%) developed PPTX. At 1 year, PPTX was associated with increased risk of all‐cause mortality (HR 16.44, 95% CI 11.16–24.23), pneumonia (HR 6.14, 95% CI 3.48–10.83), dysphagia (HR 4.86, 95% CI 3.23–7.33), vocal cord paralysis (HR 5.19, 95% CI 3.31–8.15), and need for mechanical ventilation (HR 18.13, 95% CI 10.89–30.17). PPTX was also associated with higher rates of hospital readmission (HR 15.12, 95% CI 11.97–19.10) and emergency room visits (HR 2.09, 95% CI 1.33–2.28) (all *p* < 0.01). All outcomes were also significant at 3 and 6 months after surgery.

**Conclusion:**

PPTX after TD or PT is exceedingly uncommon but associated with significantly worse mortality and morbidity.

**Level of Evidence:**

3

## Graphical abstract

Postoperative pneumothorax (PPTX) is rare after thyroidectomy or parathyroidectomy, occurring in up to 0.10% of cases. However, it is associated with increased risks of all‐cause mortality, adverse respiratory events, and healthcare utilization rates in up to 1 year after surgery, deeming PPTX as a potential marker of increased postoperative vulnerability.

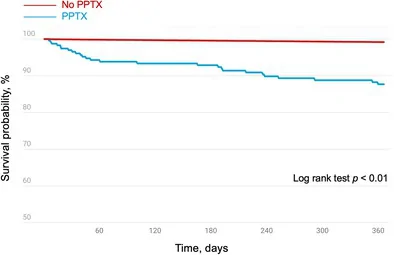

## 1 Introduction

Thyroidectomy (TD) and parathyroidectomy (PT) are among the most commonly performed endocrine surgical procedures and are generally associated with low rates of serious postoperative complications [1, 2]. Well‐recognized adverse events include hypocalcemia, recurrent laryngeal nerve injury, bleeding, and infection, most of which are routinely anticipated and promptly managed [3, 4, 5, 6]. In contrast, postoperative pneumothorax (PPTX) is an exceedingly rare complication. As a result of its rarity, PTX after thyroid or parathyroid surgery has largely been described in isolated case reports and small case series, limiting understanding of its true incidence and clinical significance.

Despite being uncommon, PPTX may represent a marker of severe perioperative physiologic insult and has the potential to precipitate respiratory failure, prolonged hospitalization, and downstream complications [7]. Existing literature offers minimal insight into whether this complication confers excess short‐term or long‐term risk beyond the immediate postoperative period. Moreover, prior studies have not systematically evaluated the association between early PPTX and subsequent morbidity, healthcare utilization, or mortality across practice settings throughout the United States.

To address this gap, we aimed to evaluate the incidence of PPTX after TD or PT and its association with mortality outcomes, postoperative complications, and healthcare utilization. By leveraging a large, multicenter dataset, this study provides the most comprehensive assessment to date of the prognostic implications of this rare but potentially devastating complication.

## 2 Methods

### 2.1 Data Source

This study employed a retrospective cohort design using the TriNetX United States Collaborative Research Network, a large, federated analytics database that continuously curates deidentified electronic health record data from over 65 participating healthcare organizations, encompassing more than 110 million patients nationwide [8]. The database captures both inpatient and outpatient clinical information, including patient demographics, diagnostic and procedural codes, laboratory results, vital signs, medication exposures, and mortality data.

Data are contributed directly by participating institutions and harmonized within a common data framework using validated mapping methodologies, natural language processing techniques, and rigorous quality control measures. To ensure patient privacy, TriNetX performs irreversible deidentification of all records in accordance with the Health Insurance Portability and Accountability Act (HIPAA), precluding patient‐level reidentification. The database supports live cohort assembly and longitudinal outcome analyses through various coding systems including the International Classification of Diseases, 9th and 10th Revision (ICD‐9 and ICD‐10, respectively), Current Procedural Terminology (CPT), and Systematized Nomenclature of Medicine (SNOMED).

### 2.2 Study Population

Using TriNetX, we conducted a real‐time database query to identify eligible patients treated between January 1, 2004, and December 1, 2025. The study population included adults aged 18 years or older who underwent open TD or PT. Patients were subsequently categorized into two groups based on the occurrence of PTX within 7 days following surgery. Individuals with missing data or inconsistent longitudinal follow‐up within TriNetX were excluded from the analysis. Surgical procedures and PPTX were identified using standardized ICD‐10, CPT, and SNOMED codes which are detailed in Table S1.

### 2.3 Study Outcomes

The primary outcome was the risk of all‐cause mortality. Secondary outcomes included various aspects of neck complications including risks of pneumonia, dysphagia, vocal cord paralysis, other respiratory complications, and need for mechanical ventilation or tracheostomy. Healthcare utilization rates, including hospital readmission rates and emergency room visits, were also assessed. To ensure accurate temporal sequencing and capture of incident outcomes, diagnoses documented prior to the defined postoperative window were systematically excluded. Detailed descriptions of the ICD‐10, CPT, and SNOMED codes used to identify study outcomes are provided in Table S2.

### 2.4 Data Analysis

Statistical analyses were conducted using the embedded analytical tools within TriNetX and further supported by R statistical software version 4.5.2 (R Foundation for Statistical Computing, Vienna, Austria) for independent verification. Comparisons of categorical variables were performed using two‐tailed Chi‐square testing. Effect estimates were expressed as odds ratios (ORs) and hazard ratios (HRs) with corresponding 95% confidence intervals (CIs) derived from Cox proportional hazards regression models. All hypothesis testing was two‐sided, with statistical significance defined as a *p*‐value < 0.05.

## 3 Results

### 3.1 Characteristics of the Study Population

Overall, 228,340 patients undergoing TD or PT were analyzed, with 237 (0.10%) patients having experienced PPTX (Table 1). The PPTX cohort had a mean follow‐up time of 295.6 ± 118.6 days, while the non‐PPTX cohort had a mean follow‐up time of 292.7 ± 130.2 days.

**TABLE 1:** Baseline characteristics of the study population.

Characteristics	PPTX (*n* = 237)	No PPTX (*n* = 228,103)	*p*
Demographics			
Age at index, SD	58.7 ± 14.4	54.6 ± 15.0	**< 0.01**
Female	128 (54.1)	169,267 (74.2)	**< 0.01**
Black or African American	50 (21.1)	33,058 (14.5)	**< 0.01**
White	131 (55.3)	148,539 (65.1)	**0.04**
Asian	12 (5.1)	9254 (4.1)	0.32
American Indian or Alaska Native	0 (0.0)	922 (0.4)	0.34
Native Hawaiian or Pacific Islander	10 (4.2)	1228 (0.5)	**< 0.01**
Not Hispanic or Latino	181 (76.4)	175,072 (76.7)	0.10
Hispanic or Latino	17 (7.2)	18,612 (8.2)	0.77
Medical comorbidities			
Diabetes mellitus	42 (17.7)	34,878 (15.3)	0.14
Nicotine dependence	44 (18.6)	20,352 (8.9)	**< 0.01**
Hypertensive diseases	116 (48.9)	85,268 (36.4)	**< 0.01**
Chronic kidney disease	34 (14.3)	18,158 (8.0)	**< 0.01**
Heart failure	25 (10.5)	9386 (4.1)	**< 0.01**
Overweight and obesity	54 (22.8)	46,007 (20.2)	0.13
Asthma	32 (13.5)	22,931 (10.1)	0.09
Emphysema	16 (6.8)	3427 (1.5)	**< 0.01**
Unspecified chronic obstructive pulmonary disease	25 (10.5)	9061 (4.0)	**< 0.01**
Preoperative diagnoses			
Thyroid cancer	60 (25.3)	35,668 (15.6)	**< 0.01**
Thyrotoxicosis	18 (7.6)	27,957 (12.3)	0.06
Nontoxic goiter	135 (57.0)	137,757 (60.4)	0.97
Hyperparathyroidism	62 (26.2)	74,257 (32.6)	0.13

*Note:* Data are reported as count (percentage). Two‐sided Chi‐square or Student's *t*‐test was used. Bold *p*‐values indicate statistical significance.

Abbreviations: PPTX, postoperative pneumothorax; SD, standard deviation.

The PPTX cohort were more likely to be older (58.7 ± 14.4 vs. 54.6 ± 15.0 years, *p* < 0.01), and Black or African American (21.1% vs. 14.5%, *p* < 0.01) or Native Hawaiian or Pacific Islander (4.2% vs. 0.5%, *p* < 0.01), while a smaller proportion were White (55.3% vs. 65.1%, *p* = 0.04). The PPTX cohort demonstrated a higher burden of comorbid disease, including nicotine dependence (18.6% vs. 8.9%), hypertensive disease (48.9% vs. 36.4%), chronic kidney disease (14.3% vs. 8.0%), heart failure (10.5% vs. 4.1%), emphysema (6.8% vs. 1.5%), and chronic obstructive pulmonary disease (10.5% vs. 4.0%) (all *p* < 0.01). Furthermore, thyroid cancer was more common among patients with PPTX (25.3% vs. 15.6%, *p* < 0.01).

### 3.2 Short‐Term Outcomes

At 3 months, PPTX was associated with an increased risk of all‐cause mortality (OR 29.33, 95% CI 16.97–50.71) (Table 2). Patients with PPTX also demonstrated higher odds of dysphagia (OR 6.15, 95% CI 3.61–10.45), vocal cord paralysis (OR 5.46, 95% CI 3.17–9.41), and the need for mechanical ventilation (OR 27.14, 95% CI 15.01–49.06). PPTX was also associated with increased hospital readmission (OR 26.90, 95% CI 19.69–36.74), while pneumonia and emergency department visits could not be assessed due to low event counts.

**TABLE 2:** Comparative 3‐month outcomes between patients with and without postoperative pneumothorax.

Outcomes	PPTX	No PPTX	*p*	OR	95% CI
All‐cause mortality	14 (5.9)	486 (0.2)	**< 0.01**	29.33	(16.97, 50.71)
Pneumonia	*	*	*	*	*
Dysphagia	15 (6.3)	3132 (1.4)	**< 0.01**	6.15	(3.61, 10.45)
Vocal cord paralysis	14 (5.9)	3075 (1.3)	**< 0.01**	5.46	(3.17, 9.41)
Need for mechanical ventilation	12 (5.1)	616 (0.3)	**< 0.01**	27.14	(15.01, 49.06)
Hospital readmission	68 (28.7)	5225 (2.3)	**< 0.01**	26.90	(19.69, 36.74)
Emergency room visits	*	*	*	*	*

*Note:* Data are reported as count (percentage). Two‐sided Chi‐square or Student's *t*‐test was used. Bold *p*‐values indicate statistical significance *p* < 0.05. Asterisks indicate counts too small (< 10) to compute.

Abbreviations: CI, confidence interval; PPTX, postoperative pneumothorax; SD, standard deviation.

At 6 months, PPTX remained significantly associated with increased mortality (OR 19.38, 95% CI 11.61–32.34) (Table 3). Increased odds persisted for dysphagia (OR 6.08, 95% CI 3.81–9.71), vocal cord paralysis (OR 5.21, 95% CI 3.12–8.70), and mechanical ventilation (OR 23.14, 95% CI 13.09–40.91). Hospital readmission (OR 22.88, 95% CI 16.76–31.23) and emergency department utilization (OR 2.39, 95% CI 1.38–4.14) also remained higher, while pneumonia still could not be assessed due to low event counts.

**TABLE 3:** Comparative 6‐month outcomes between patients with and without postoperative pneumothorax.

Outcomes	PPTX	No PPTX	*p*	OR	95% CI
All‐cause mortality	16 (6.8)	847 (0.4)	**< 0.01**	19.38	(11.61, 32.34)
Pneumonia	*	*	*	*	*
Dysphagia	20 (8.4)	4333 (1.9)	**< 0.01**	6.08	(3.81, 9.71)
Vocal cord paralysis	16 (6.8)	3713 (1.6)	**< 0.01**	5.21	(3.12, 8.70)
Need for mechanical ventilation	13 (5.5)	787 (0.3)	**< 0.01**	23.14	(13.09, 40.91)
Hospital readmission	69 (29.1)	6266 (2.7)	**< 0.01**	22.88	(16.76, 31.23)
Emergency room visits	14 (5.9)	6406 (2.8)	**< 0.01**	2.39	(1.38, 4.14)

*Note:* Data are reported as count (percentage). Two‐sided Chi‐square or Student's *t*‐test was used. Bold *p*‐values indicate statistical significance *p* < 0.05. Asterisks indicate counts too small (< 10) to compute.

Abbreviations: CI, confidence interval; PPTX, postoperative pneumothorax; SD, standard deviation.

### 3.3 Long‐Term Outcomes

**FIGURE 1:**
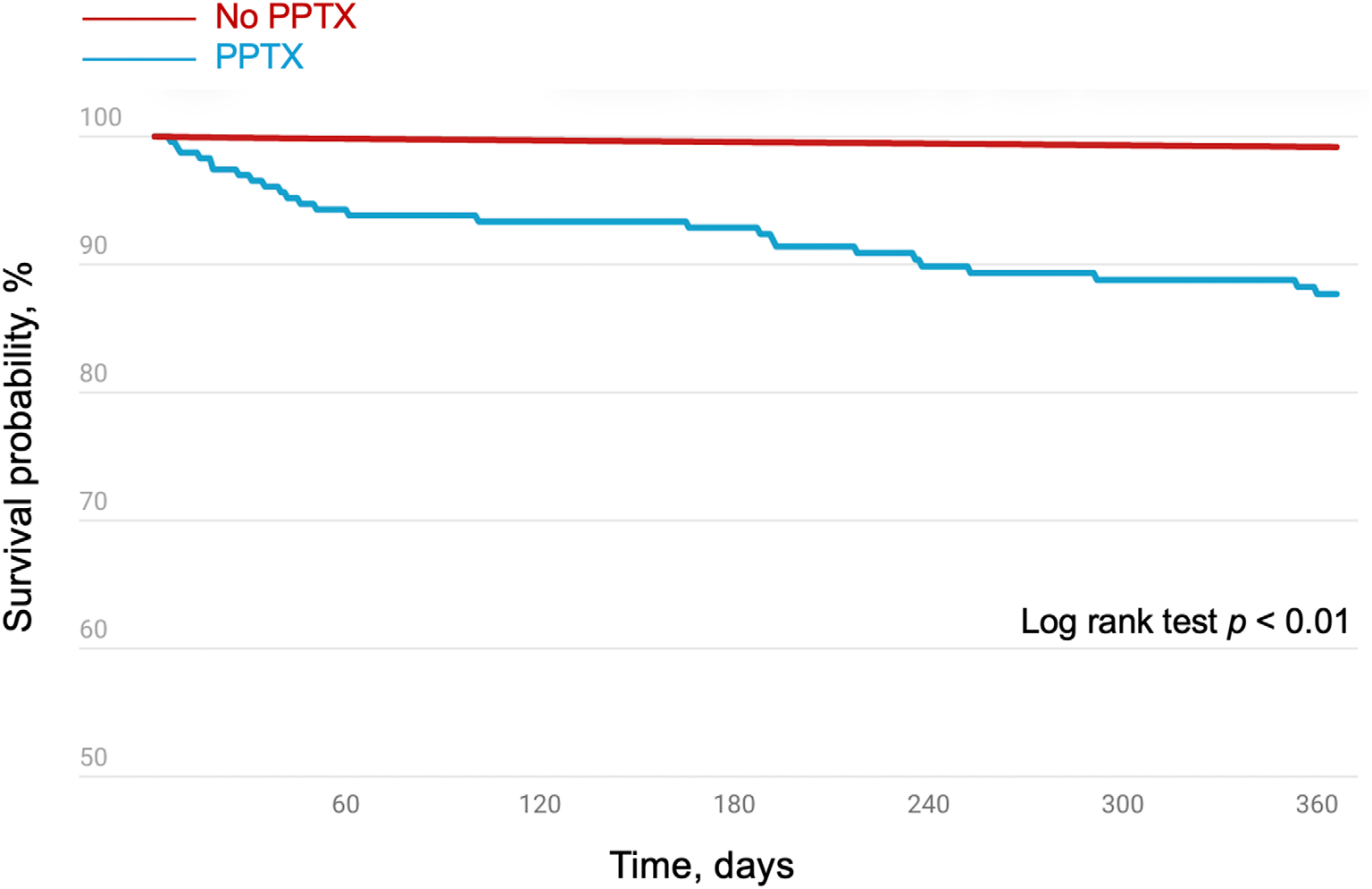
Kaplan–Meier curves for all‐cause mortality between the presence and absence of postoperative pneumothorax after surgery over 1 year of follow‐up. [Color figure can be viewed in the online issue, which is available at www.laryngoscope.com]

At 1 year, PPTX was associated with an increased risk of all‐cause mortality (HR 16.44, 95% CI 11.16–24.23) (Table 4). Indeed, Kaplan–Meier survival analysis demonstrated significantly worse survival among patients who developed PPTX compared with those without PPTX (Figure 1).

**TABLE 4:** Comparative 1‐year outcomes between patients with and without postoperative pneumothorax.

Outcomes	PPTX	No PPTX	*p*	HR	95% CI
All‐cause mortality	26 (11.0)	1516 (0.7)	**< 0.01**	16.44	(11.16, 24.23)
Pneumonia	12 (5.1)	2188 (1.0)	**< 0.01**	6.14	(3.48, 10.83)
Dysphagia	23 (9.7)	5924 (2.6)	**< 0.01**	4.86	(3.23, 7.33)
Vocal cord paralysis	19 (8.0)	4133 (1.8)	**< 0.01**	5.19	(3.31, 8.15)
Need for mechanical ventilation	15 (6.3)	1090 (0.5)	**< 0.01**	18.13	(10.89, 30.17)
Hospital readmission	71 (30.0)	7803 (3.4)	**< 0.01**	15.12	(11.97, 19.10)
Emergency room visits	19 (8.0)	9349 (4.1)	**< 0.01**	2.09	(1.33, 2.28)

*Note:* Data are reported as count (percentage). Two‐sided Chi‐square or Student's *t*‐test was used. Bold *p*‐values indicate statistical significance *p* < 0.05.

Abbreviations: CI, confidence interval; HR, hazards ratio; PPTX, postoperative pneumothorax.

Patients with PPTX also demonstrated higher risks for all secondary outcomes including pneumonia (HR 6.14, 95% CI 3.48–10.83), dysphagia (HR 4.86, 95% CI 3.23–7.33), vocal cord paralysis (HR 5.19, 95% CI 3.31–8.15), and need for mechanical ventilation (HR 18.13, 95% CI 10.89–30.17). Regarding healthcare utilization, PPTX was associated with increased hospital readmission (HR 15.12, 95% CI 11.97–19.10) and emergency department utilization (HR 2.09, 95% CI 1.33–2.28).

## 4 Discussion

In the present study, we discovered the incidence of PPTX after TD or PT to be 0.10% which is greater than prior reports of 0.02%, although documented reports are scarce and may underscore this degree of rarity [9]. We also observed markedly higher short‐term adverse events and persistently elevated 1‐year hazards of mortality and major respiratory complications, alongside increased healthcare utilization rates. It is important to note that the low absolute counts of PPTX relative to the inclusion period may explain the abnormally high OR and HR values as well as wide variability of CIs, warranting very special caution when interpreting the findings. Nevertheless, the clear and consistent associations between PPTX after TD or PT and the mortality and morbidity outcomes observed in this analysis demonstrate that even when infrequent, this complication carries a large prognostic signal at scale.

PPTX after open TD or PT is very likely related to various surgical variables, especially those that involve occult pleural injury during deep dissection near the thoracic inlet, superior mediastinal exploration, or air leak from cervical fascial planes into the mediastinum with subsequent pleural rupture [10, 11]. Indeed, procedural events and operative complexity are key contributors, although these factors could not be directly assessed in this analysis. Although there is limited background literature investigating this, published cases of PPTX have described this complication occurring after TD and PT individually, suggesting that pleural compromise during surgery is not limited to a single cervical endocrine procedure. Eltzschigm Posner, and Moore [12] attributed their observed PPTXs following TD to pleural punctures in the neck exacerbated by the negative pressure of spontaneous ventilation, which was evident in two cases of infraclavicular jugular node dissections. Guerrero et al. [13] described extreme neck hyperextension during PT as a commonality in their case series of four PPTXs after the procedure was performed in a minimally invasive manner, with additional risks including superior mediastinal dissection, traction on the thyrothymic ligament, low‐lying inferior parathyroid glands, and general use of electrocautery which can inadvertently injure the pleura. Slater and Inabnet [14] attributed their two cases of PPTX to either the close proximity of parathyroid adenoma to the lung pleura or patient‐specific history, namely emphysema which is associated with pleural blebs which may have ruptured during the procedure. Collectively, these associations may remind surgeons of the importance of intraoperative awareness and early postoperative recognition of respiratory symptoms in patients who underwent more extensive dissection or structural irritation.

In particular, extended resections involving nearby organs can bring the surgical field much closer to pleural structures, increasing the risk for PPTX. In the context of thyroid cancer, cases can frequently involve extensive surgery and neck dissection, with high‐risk cases involving local invasion to the larynx or trachea potentially requiring extensive segmental resection [15, 16, 17]. Specifically, injury to these structures can exist either from liberating the isthmus from the trachea or from dissecting the posterior‐lateral aspect of the trachea in the region where the recurrent laryngeal nerve meets the tracheal cartilage [18]. In the context of primary hyperparathyroidism requiring PT, there may exist special circumstances that warrant assistance from thoracic surgeons, such as thyroid glands extending far below the sternal notch or measuring widely in the horizontal dimension requiring thoracic exposure [19]. Furthermore, concomitant thymectomy can commonly occur, although this may further increase the risk for PPTX as it not only requires dissection into the superior mediastinum where the pleura is thin and closely attached to thymic tissue, but also allows for further mediastinal exploration after thymus removal and increases the probability for iatrogenic pleural injury [13, 20, 21]. Whereas PPTX arises from air entry into the mediastinum from this pleural compromise, pneumomediastinum is another related but anatomically different complication that involves air entry into the mediastinum specifically from tracheal or esophageal perforation along the cervical fascial planes which can lead to subcutaneous emphysema and neck swelling in a majority of cases [22, 23]. Regarding management, PPTX generally requires chest tube placement while pneumomediastinum in this context may resolve with conservative management, with surgical intervention warranted if the condition persists, worsens, or if there is a high risk of necrosis or acute airway compromise [14, 15, 23, 24, 25].

While the aforementioned surgical factors are central, the differences in baseline comorbidities observed in this analysis suggest that patient‐level vulnerability may also contribute meaningfully to the risk of developing PPTX. For instance, the PPTX cohort had substantially higher rates of some chronic obstructive pulmonary disease and nicotine dependence, suggesting limited pulmonary reserve and greater susceptibility to barotrauma under positive‐pressure ventilation which can cause an air leak [26]. However, PPTX in these high‐risk patients is rarely an isolated radiographic finding in practice. Rather, it can precipitate hypoxemia, escalation of ventilatory support, and prolonged critical care trajectories in the most severe cases. Indeed, new‐onset dyspnea or increasing oxygen requirements after either TD or PT may warrant chest radiography in such patients to rule out PPTX, although there is a low threshold for computed tomography which has been increasingly used for surgically complex cases [27]. Given the magnitude of the risk for respiratory failure and mortality that was observed, it is reasonable to consider protocols emphasizing not only early imaging, but also respiratory therapy engagement and appropriate discharge planning with close outpatient follow‐up [28]. Nevertheless, it is important to note that the risk of developing PPTX is not solely procedure‐driven, but also reflects underlying patient morbidities and individualized risk profiles which can assist with perioperative risk stratification and monitoring.

This study has several limitations which are inherent to retrospective analyses using large administrative databases like TriNetX. These include the potential for miscoding or misclassification of diagnoses and procedures, incomplete capture of events occurring in HCOs not included in this analysis, and limited granularity regarding intraoperative technique and perioperative management. In addition, the database did not provide PTX‐specific clinical details including but not limited to laterality, size, or the type of intervention performed (e.g., observation, needle decompression, chest tube placement), all which can meaningfully influence interpretation. Finally, due to small event counts, we were unable to stratify outcomes by TD versus PT, assess the extent of cervical surgery, or evaluate the impact of concomitant neck dissection or mediastinal exploration in malignant cases. The small number of cases observed may also explain the wide magnitudes of ORs and HRs as well as the breadths of their CIs, requiring careful interpretation. Nevertheless, this study represents the largest population‐based analysis to date that examines PPTX after either of the two procedures. Given the rarity of this complication and significant magnitudes of the observed risk estimates, these findings should be taken seriously but also with appropriate caution.

## 5 Conclusion

In this nationwide analysis, PPTX occurring shortly after open TD or PT was associated with substantially increased mortality, respiratory complications, and healthcare utilization. This is a very rare complication that may explain the drastic magnitudes of effect estimates and the width of CIs, requiring cautious and balanced interpretation of the results. Nevertheless, these findings suggest that PPTX may serve as a marker of increased postoperative vulnerability, particularly in patients with significant pulmonary illness or malignant disease requiring extensive dissection. Perioperative vigilance and early surveillance may be warranted in affected patients.

## Funding

The authors have nothing to report.

## Ethics Statement

Ethical review and approval were waived for this study.

## Consent

Patient consent was waived since it was retrieved from a database which contains de‐identified data.

## Conflicts of Interest

The authors declare no conflicts of interest.

## Supporting information


**Table S1:** Diagnostic codes for study inclusion criteria.
**Table S2:** Diagnostic codes for study outcomes.

## Data Availability

De‐identified datasets were analyzed in this study. These data can be found at https://trinetx.com.
