# Supplementary material for: Rare But Risky: Clinical Impacts of Pneumothorax After Thyroidectomy and Parathyroidectomy
Source: Laryngoscope. 2026 Jun 12;136(10):4589–95. doi: 10.1002/lary.70646 (PMC13569608; doi:10.1002/lary.70646)
Supplement: Supplementary file 1 [file LARY-136-4589-s001.docx]

**Supplementary Table 1**. Diagnostic codes for study inclusion criteria.

| **Procedure** | **ICD-10 Code** | **CPT Code** | **SNOMED Code** |
| --- | --- | --- | --- |
| Partial/total thyroidectomy | –– | 60210, 60212, 60220, 60225, 60240, 60252, 60254, 60270, 60271, 1009035 | 13619001,  237486002, 24443003, 24711004,  302338000, 30956003,  359882009, 52826006,  53533006 |
| Parathyroidectomy | 0GBR0ZZ | 1014226 | 53304009 |

ICD: International Classification of Diseases; CPT: Current Procedural Terminology: SNOMED: Systematized Nomenclature of Medicine. Dashes indicate coding system not utilized.

**Supplementary Table 2**. Diagnostic codes for study outcomes.

| **Outcome** | **ICD-9 Code** | **ICD-10 Code** | **CPT Code** | **SNOMED Code** |
| --- | --- | --- | --- | --- |
| All-cause mortality | * | * | * | * |
| Pneumonia | 483 | J12-J18 | –– | –– |
| Dysphagia | –– | R13.1 | –– | –– |
| Vocal cord paralysis | –– | J38.0 | –– | –– |
| Need for mechanical ventilation | 39.65 | 5A1945Z, 5A1955Z, 0BH13EZ, 0BH17EZ, 0BH18EZ | 31500, 1022227 | 1015098 |
| Hospital readmission | –– | –– | 1013668 | –– |
| Emergency room visits | * | * | * | * |

ICD: International Classification of Diseases; CPT: Current Procedural Terminology: SNOMED: Systematized Nomenclature of Medicine. Dashes indicate coding system not utilized. Asterisks indicate intrinsically coded within TriNetX.
